# Opinions of married women about potential causes and triggers of intimate partner violence against women. A cross-sectional investigation in an Iranian city

**DOI:** 10.1186/1471-2458-8-209

**Published:** 2008-06-12

**Authors:** Behrooz Hamzeh, Mirtaghi Garousi Farshi, Lucie Laflamme

**Affiliations:** 1 Division of International Health, Department of Public Health Sciences, Karolinska Institutet, Nobels väg 9, SE-171 77 Stockholm, Sweden; 2 Kermanshah University of Medical Sciences, Kermanshah, Islamic Republic of Iran; 3 National Public health Management Centre (NPMC), Tabriz University of Medical Sciences, Tabriz, Islamic Republic of Iran; 4 Faculty of Education and Psychology, Department of Psychology, Tabriz University, Tabriz, Islamic Republic of Iran

## Abstract

**Background:**

Population-based perception studies on potential causes and triggers of intimate partner violence against women (IPVAW) may enlighten context-relevant primary preventive actions in settings where data are limited. This study, conducted in one specific city, deals with married women's opinions concerning potential causes and triggers of IPVAW and seeks to highlight areas of consensus and divergence in the views.

**Methods:**

A convenient sample of women aged 25–45 years and married for at least 5 years was consecutively recruited in the 48 public health centres of Kermanshah city, where free health services are provided to mothers and children under 6 years old. Respondents were individually interviewed on site by trained and experienced female interviewers (response rate 94.3%). A structured pilot-tested questionnaire was used that consisted mainly of closed questions about individual assessment of the extent to which various items could be regarded as a potential cause, a potential trigger or a potential consequence of IPVAW. Individual item frequencies were compiled and the association between socio-demographic attributes of the spouses and also respondents' prior exposure to violence and women answers was explored.

**Results:**

For most factors covered, women mainly "agreed" or "agreed very much" about their potential as a trigger or a cause of IPVAW; agreements were stronger for individual-related potential causes. Generally, women's socio-demographic characteristics and prior victimisation did not much affect the opinions they expressed. For some triggers however, women's own occupation and their husband's educational level affected how much in agreement they were.

**Conclusion:**

The women interviewed consider that most potential causes and triggers proposed may, at some point in a relationship, engender IPVAW. In the main, their views are not much altered by their own and their husbands' socioeconomic position or their prior victimisation. It remains to be seen whether married men and, for that matter, even women married for a shorter duration or from other settings will answer in a similar manner.

## Background

Domestic violence is acknowledged as the most common and hidden form of violence against women [1], one of the most prevalent forms being intimate partner violence against women (IPVAW) [2,3]. International definitions of IPVAW encompass a range of coercive acts and behaviours of a physical, sexual and psychological nature which are used against them by their current or former intimate partners [4,5]. Yet, a universally accepted definition remains difficult to reach given cultural or societal diversity both between and even within countries [6,7]. Indirect and verbal aggression between intimate partners for instance is not always considered as an act of violence – or not always covered in empirical studies [8].

IPVAW affects women from every cultural, social and economic group [2,5,9]. Prevalence studies from various countries relying on population-based surveys show that from 10% to over 69% of women may experience physical violence by a male partner during their life [5,10]. Beside it being prevalent, IPVAW has wide-ranging short- and long-term effects on the victims themselves, on their offspring and on the quality of their relationship with their partner [2,5,11].

Epidemiologic studies have helped to highlight a series of potential risk factors of IPVAW, and their classification into remote and triggering factors is quite well established, the latter having rather immediate effects on the perpetration of acts of violence and the former (causes) forming part of the distal background factors of the phenomenon [5,12]. The ecological model widely used in public health settings classifies the various – and interacting – domains lying behind those factors in individual, familial/relational, local community and society as a whole [2,13]. Documented examples of *individual *potential causes are young age of the perpetrator [reviewed in [14]] and alcohol consumption [10,14-18]; marital instability [10,14,16,18] and economic stress [5] are acknowledged *family-related *potential causes. Supportive social norms, weak sanctions against perpetrators, low social capital, traditional gender norms and poverty are considered as *societal and community-related *potential causes [5,18].

The ecological approach stresses the importance of taking into account the global context of occurrence of IPVAW in order to better understand why it occurs and – perhaps more importantly – how preventive efforts can be tailored to different groups. In that respect, the perception of various community members about the potential causes and triggers of IPVAW could constitute an important part of preventive efforts, in particular in settings where knowledge is limited and data are scarce and difficult to access [19]. Indeed, prior to epidemiologic investigation and intervention, performing social diagnosis about potential causes and triggers of IPVAW can help to clarify a number of beliefs as well as consensus and divergence about them within and between groups of people [19,20].

This paper presents the results of a social diagnosis investigation gathering the views of married Iranian women from Kermanshah city concerning what may cause or trigger IPVAW. The study forms part of a broader community-based project ultimately aiming at the prevention of IPVAW in new couples and of which the first part is a wide-ranging social assessment that gathers the opinions of women and men currently married as well as those of a number of key informants in the community so as to better understand the social context of the intervention that will be developed later on. The approach is inspired from the Green model for the modification of health behaviour [developed in 1991] and where various community-specific assessments ought to be made prior to an intervention and the first step is a social assesment [21,22].

A wide range of factors (and potential beliefs) are covered, including both attitudes and values that may need to be altered and are modifiable in an educational programme helping new married couples to reflect upon their attitudes and also solve their conflicts in a non-violent and non-aggressive manner. This approach felt necessary as Iranian studies on this topic and on IPVAW in general are still limited and scattered [23-27]. Also, as physical IPVAW is illegal, reports are few; although governmental victim services are available. In the survey reported herein, the main question was how married women from the city assess documented potential causes and triggers of IPVAW? Complementarily attention was paid to their views about a number of consequences. For explorative purposes, the association between socio-demographic attributes of the spouses and women opinions was considered. Even the association with disclosed exposure to violence was looked into.

## Methods

### Setting, sample and case recruitment

Kermanshah city, where the study was conducted, is the capital of the province Kermanshah and has a population of about 800,000 inhabitants. In the city, the three main ethnic groups called the Kurds (in majority), the Fars and the Laks (minority) cohabit. The Kurds, who also live in Iraq and Turkey, and the Laks live only in the western part of Iran – where Kermanshah is situated – and the Fars are in the majority in most of the rest of the country. Kermanshah is considered to have a patriarchal culture stronger than that of many other Iranian cities and the divorce rate is 3.4% [28].

The interviews were conducted during August 2005 in the city of Kermanshah, seeking married women of reproductive age co-habiting with an intimate male partner. As the average age of marriage for women in the city is 20.3 years [reference year 2000; [28]], women aged 25–45 years, currently married and with at least 5 years of marriage behind them were approached when they visited any one the of city's 48 public health centres. These centres all provide free health care to mothers and children under 6, e.g. vaccines, contraceptives, and visits to the doctor and midwife. According to reports by Kermanshah district health centre, 80% of married women of reproductive age visit the centres for different purposes each year. As women usually visit the centres unaccompanied by their husband, they were regarded as both effective and safe recruitment sites [29,30].

The sample size was estimated at 460 women, considering the Cochran formula allowing about 15% attrition. Interviews were conducted in all 48 centres, in proportion to each centre's population coverage. Women were recruited consecutively, on site, by trained interviewers (see below). In total, 435 of the 460 eligible women approached were interviewed (non-response rate: below 6%); none of them was accompanied by her husband.

### Data collection instruments and strategy

A questionnaire in five parts consisting mainly of closed questions was developed and pre-tested. Part I covered the spouses' socio-demographics and Part II addressed women's prior exposure to IPVAW. Here, a set of questions focusing on non-physically abusive acts including moderate forms of violence [31], like shouting and physical threats, was used on the grounds that physical IPVAW is illegal in Iran and interviewed women may feel more comfortable if not directly questioned on such matters (that were not otherwise the main focus of the study). Part III and IV considered, in turn, potential causes and triggers of IPVAW and potential consequences. Part III proposed a list of 29 well-documented individual and relational both potential causes and triggers of IPVAW (see lists in Tables 1 and 2), often referred to in the international literature [5] and some others raised either during the pre-test with men and women or during the following discussion within the research group: "the wife putting on unsuitable clothes in front of others", "saying unpleasant things in front of others", "questioning husband's friends", and "disagreement about leisure time activity". Part IV, structured in the same manner as part III introduced seven individual and familial consequences of IPVAW including e.g., psychological problems for the wife, children and husband as well as learning problems of children at school (see list in Table 3). Physical consequences were not addressed as we were expecting very high consensus among women regarding them. The interview ended with a few questions relative to the prevention of IPVAW (Part V).

**Table 1:** Participants' opinions about 13 potential triggers of IPVAW. Items ranked in descending order of "complete agreement"

	% respondents' opinions*				
	
**Potential triggers**	**1**	**2**	**3**	**4**	**5**
The wife saying unpleasant things to husband in front of others	88.5	7.1	0.9	0.9	0.2
The wife putting on unsuitable clothes in front of others	86.9	8.3	0.7	1.6	0.5
The husband suspecting the wife of infidelity	75.9	12.4	2.5	5.3	1.6
The wife arguing back	72.9	20.5	1.6	2.5	0.9
The wife not obeying the husband	69.2	23.2	1.6	3.4	0.9
Quarrelling about each other's family	65.7	24.1	3.4	3.9	0.7
The wife not caring adequately for the children	44.8	41.8	2.5	7.4	1.8
The wife not having food ready on time	44.6	32.0	2.1	14.7	5.1
The wife suspecting the husband of infidelity	42.1	31.5	4.4	14.9	5.5
The wife not taking good care of the home	41.4	38.6	3.2	11.0	4.1
The wife questioning the husband about money	38.9	29.4	3.4	16.1	10.6
The wife questioning the husband about his friends	23.0	21.8	5.1	23	25.1
Disagreement about leisure time activities	14.3	20.7	4.6	21.6	36.8

* 1, 2, 3, 4 and 5 are "completely agree", "agree", "no idea", "disagree" and "completely disagree" respectively.

**Table 2:** Participants' opinions about 16 potential causes of IPVAW. Items ranked in descending order of "complete agreement"

	% respondents' opinions*				
	
**Potential causes**	**1**	**2**	**3**	**4**	**5**
Addiction of a partner	92.2	4.8	0.7	1.1	0.5
Mental disorder of a partner	88.7	8.5	0.5	0.9	0.0
Husband's long term unemployment	83.0	10.3	2.3	3.2	0.0
Unsatisfying sexual relationship	75.9	12.2	3.0	6.9	0.5
Difficulties at husband's work	62.1	20.5	2.3	11.5	0.3
Large age difference between husband and wife	53.3	29.0	2.8	11.5	2.3
Unsolved marital conflict	53.3	27.1	4.8	12.0	2.1
Economic problems in the household	45.7	30.6	1.6	18.6	2.3
Difference in social class between husband and wife	45.3	28.7	5.5	16.6	2.8
Poor verbal communication between husband and wife	43.7	33.8	3.7	10.3	7.6
Marital instability (threat to survival of the marriage)	40.2	30.1	6.2	18.9	3.9
Young age of the partners	32.9	31.5	5.1	22.8	6.7
Difficulties in raising children	29.7	44.1	3.7	16.6	4.4
Absence of male child	26.0	17.9	6.0	26.4	22.8
Amount of dowry/mahrieh	15.2	23.2	4.6	24.4	31.0
Disagreement about the share of housework	12.9	18.4	5.5	22.8	38.9

* 1, 2, 3, 4 and 5 are "completely agree", "agree", "no idea", "disagree" and "completely disagree" respectively.

**Table 3:** Participants' opinions about 7 potential consequences of IPVAW. Items ranked in descending order of "complete agreement"

	% respondents' opinions*				
	
**Potential consequences**	**1**	**2**	**3**	**4**	**5**
Mental problems of children	81.4	12.4	1.1	0.9	0.2
Long-lasting mental problems for the wife	80.2	14.0	0.2	1.1	0.5
Learning problems of children at school	69.9	22.3	1.8	1.6	0.5
Inability of the wife/mother to look after her children and family matters	67.4	23.0	1.4	3.9	0.5
Husband's mental problem	63.2	19.5	2.5	6.9	3.9
Marital instability (threat to the survival of the marriage)	61.8	25.7	2.8	4.1	1.6
Economic problems	25.1	28.7	6.4	19.1	16.8

* 1, 2, 3, 4 and 5 are "completely agree", "agree", "no idea", "disagree" and "completely disagree" respectively

For each single item presented in Part III and Part IV, respondents were asked to state the extent to which they agreed that it could cause (or trigger) the occurrence of IPVAW alternatively be a consequence of its occurence. The answer alternatives were: completely disagree, disagree, don't know, agree and completely agree.

Three trained female interviewers, all of which were experienced and university graduates (BSc degree) collected the data consecutively and in a convenient manner. Interviews were conducted privately at each health centre on every day of the week. Upon arrival at the centre, women were asked by the interviewer whether they would be willing to participate in the study. They were informed about the study goals, briefed about the content of the questionnaire, received assurance of confidentiality and notified of the possibility to withdraw from the interview at any time.

### Data treatment

Univariate frequencies for each response alternative were compiled and arranged in descending order of agreement (alternative 5), all respondents aggregated (using SPSS version 14). Also, item by item, comparisons were made between groups of respondents considering selected socio-demographics (marriage duration, number of children (sons and daughters), and spouses' ethnic group), education level and employment, and also prior exposure to violence. Two by two comparisons were made and the significance of the difference was estimated with 95% confidence intervals, using a test for two independent proportions (VassarStats: Web Site for Statistical Computation).

The study was reviewed and approved by the Iranian National Ethics Committee of Medical Research.

## Results

### Potential causes and triggers

The 13 potential triggers presented in the questionnaire are listed in Table 1 in descending order of the proportions of women who "agreed completely" that they were triggers of IPVAW. Several of them met with very strong consensus, with 6 being completely agreed on by more than 50% of the respondents and 11 being either agreed on or completely agreed on.

Over 80% of the women completely agreed with the triggering effect of the first two items, both of which can imply a husband losing face in front of others: unpleasant verbal communication and unsuitable clothes (88.5% and 86.9% respectively). Items related to home chores and household matters (care of child and home, and meals) score 80% when both "agree completely" and "agree" are considered. Lowest on the list are potential triggers related to leisure time activities and, otherwise, women asking questions about husband's friends. For those items, the proportion of women having "no idea" is also the highest.

Table 2 deals with potential causes of IPVAW in a similar manner to that above. Over 75% of the respondents completely agreed on four items. Of those, the first two have to do with the partner's mental health and attain very high consensus: "addiction of a partner" (92.2%) and "mental disorder of a partner" (88.7%). They are followed by husband's unemployment (83%) and sexual relationship (75.9%). It can be underlined that, "addiction of a partner" and "husband's unemployment" ranked first when women were asked to rank the top two causes or triggers of IPVAW. Absence of male child (26%), dowry (15.2%), and share of housework (12.9%) attain the lowest rates of complete agreement – and the highest rates of complete disagreement.

### Consequences

Table 3 orders the potential consequences of IPVAW according to the consensus attained on their likelihood of occurrence. Not only do children's and the wife's mental problems attain the highest rates of complete agreement (81.4% and 80.2% respectively) but they also come first when women are asked to freely order the most important consequences. All items attained over 80% of agreement (including completely agree and agree) apart from "economic problems".

### Comparisons between opinions expressed by groups of women

Table 4 presents the socio-demographic characteristics of the participants and also their own prior experience of non-physical IPVAW. Exposure to verbal abuse in the form of shouting is reported by one of two women and ranks first (52.2%); destroying property is less common (14.7%). 319 women reported moderate forms of IPVAW and victimisation. Of those, 11.6% reported only verbal abuse, 19.5% only verbal or physical threats (e.g. slamming doors) and 28.5% only nonverbal abuse (e.g. acting like " I have nothing to lose"). In an open part of the interviews, when asked to report voluntarily on their own exposure to IPVAW, 54% didn't specify anything. Among those who mentioned something, 44% reported their husband refusing to talk to them.

**Table 4:** Respondents' socio-demographic characteristics and experience of moderate intimate partner violence* (n = 435)

Characteristics	Groups	%	
	
Age (years)	25–29	39.5	
	30–34	30.1	
	35–39	17.5	
	40–45	12.9	
Marriage duration (years)	5–9	44.1	
	10–14	22.3	
	15–19	17.9	
	19+	15.4	
Situation of job	Housewife	85.7	
	Employed or student	14.3	
Education level	Illiterate, primary and guidance school (up to 8th grade)	54.5	
	High school (9th to 12th grade)	32.0	
	Under- and postgraduate	13.3	
Education level of husband	Illiterate, primary and guidance school (up to 8th grade)	41.6	
	High school (9th to 12th grade)	36.6	
	Under- and postgraduate	21.6	
Ethnicity	Kurd	65.5	
	Fars	20.0	
	Lak	12.6	
	Others	1.8	
Kind of violence			% yes in all respondents
Shouting at the respondent by her husband			52.2
Being afraid by the way husband looks at the respondent or gestures			50.2
Slamming the doors at home by respondent's husband			25.3
Acting like "I have nothing to lose" by respondent's husband			20.9
Destroying property by the respondent's husband			14.7

* Which may not be considered as "violence" in the study area.

Table 5 introduces the potential triggers, causes and consequences of IPVAW for which at least 50% of the respondents completely agreed (6, 7 and 6 items respectively), presented in descending order of complete agreement, all respondents aggregated. The proportions of complete agreement when women are stratified based on their own experience of IPVAW and on selected socio-demographic characteristics are given in the following columns. For each individual characteristic considered, significant differences in proportions are marked in bold and with the following symbols *°. Items which have significant differences with more than one other item are underlined.

**Table 5:** Percentages of the complete agreement on items reaching > 50% complete agreement by group of respondents

**Potential triggers, causes and consequences**	All	Experience of non-physical violence^a^		Occupation		Husband's education			Husband's ethnicity		
					
		Yes	No	House wife	Work or study	Up to grade 8	High school	Graduate	Kurd	Fars	Lak
**Number of respondents**^b^	**435**	**319**	**115**	**373**	**62**	**181**	**159**	**94**	**294**	**73**	**56**

**Triggers**											

Unpleasant verbal communication	88.5	89.3	87.0	89.0	85.5	89.0	89.3	86.2	87.4	87.7	94.6
Unsuitable clothes	86.9	88.1	84.3	88.7*	75.8*	90.1*	86.8	80.9*	85.7	89.0	91.1
Suspicion of infidelity	75.9	77.4	72.2	76.9	69.4	78.5	74.8	72.3	75.2*	**69.9°**	87.5*°
Arguing back	72.9	**76.5°**	**63.5°**	75.1*	59.7*	80.7*	**73.6°**	57.4*°	71.8	78.1	73.2
Not obeying	69.2	**72.7°**	**59.1°**	71.8*	53.2*	77.9*	**68.6°**	54.3*°	68.4	75.3	69.6
Quarrel about in-laws	65.7	**69.6°**	**55.7°**	67.8*	53.2*	67.4*	**70.4°**	55.3*°	65.0	71.2	66.1

**Causes**											

Addiction	92.2	92.8	91.3	92.8	88.7	93.4	91.8	90.4	93.2	90.4	91.1
Mental disease	88.7	**91.2°**	**82.6°**	90.1*	80.6*	89.5	87.4	89.4	89.8	86.3	85.7
Unemployment	83	83.4	82.6	83.4	80.6	82.9	84.9	79.8	82.0	90.4	78.6
Unsatisfying sexual relationship	75.9	74.6	80.0	76.7	71.0	77.9	76.1	72.3	73.1	80.8	82.1
Husband's work problems	62.1	63.9	57.4	64.9*	45.2*	66.3*	**67.9°**	44.7*°	61.6	65.8	57.1
Age difference	53.3	54.5	50.4	54.7	45.2	56.9	52.2	48.9	51.4	61.6	51.8
Marital conflict	53.3	54.2	51.3	51.7	62.9	53.0	52.2	55.3	52.7	60.3	53.6

**Consequences**											

Mental problems of children	81.4	82.1	80.0	83.1*	71.0*	84.5*	**83.0°**	72.3*°	81.3	86.3	75.0
Mental problems of wife	80.2	81.8	76.5	82.3*	67.7*	84.5*	**81.8°**	69.1*°	80.6	82.2	78.6
Learning problems of children	69.9	69.9	70.4	71.0	62.9	74.6	67.9	63.8	68.4*	82.2*°	**58.9°**
Inability of wife/mother to do her duties	67.4	68.7	64.3	70.8*	46.8*	71.8*	**71.7°**	51.1*°	68.7	68.5	62.5
Mental problems of husband	63.2	**66.5°**	**54.8°**	63.3	62.9	61.3	64.2	64.9	62.6	71.2	57.1
Marital instability	61.8	64.6	54.8	63.3	53.2	61.3	66.0	55.3	62.2	67.1	55.4

a) Which may not be considered as violence in the study area.

b) Number of respondents is not equal to 435 in some sub-groups because of missing answers.

*° figures with each of these symbols have significant differences with each other in their sub-group (they are in bold fonts).

The order of the items from the three parts (causes, triggers and consequences) is quite consistent among the sub-groups considered, but with some noteworthy differences. Among the most consensual potential triggers, all but the first one, "unpleasant verbal communication", show some significant inter-group differences. Also, women's occupation and husband's education level are descriptors where substantial differences are observed: housewives' scores are systematically higher than those of women who work/study. Furthermore, women whose husbands are less educated have higher scores than those whose husbands are university graduates (see for instance "arguing back"). There are also some differences based on the husband's ethnic group, particularly for "infidelity", where the consensus is higher among Laks' wives than Kurds' or Fars'. For arguing back, not obeying and quarrelling about in-laws, consensus is higher among women who experienced violence.

Inter-group differences are not very pronounced as regards the potential causes of IPVAW. When significant, they are similar to the differences found for potential triggers. Two items divide the respondents when comparing housewives and women working/studying: mental disorder of the husband and his work-related problems. Husband's work problems also score differently when women are stratified based on their husband's education.

The categories of women's occupation and husband's education also divide the respondents when it comes to the consequences of IPVAW, in particular inability of the wife to do her duties. More women married to Fars are in complete agreement as regards "children's learning problems" being a possible consequence than those married to Kurds or Laks. Also, for this item, women who have experienced violence scored higher than those who have not.

## Discussion

### Main findings

It has been stated that the prevention of IPVAW requires a community-wide response to individual risk factors, personal relationship, and larger cultural, societal and economic factors [1,32]. Although community-wide interventions on the prevention of IPVAW may rest on evidence-based strategies, the manner in which they are conducted, their content, and their mode of implementation also need to be contextualised. This study, forming part of a wider community-based social assessment, represents an attempt in that direction. It gathers opinions of married women regarding already documented potential causes and triggers of IPVAW in one specific city located in western Iran where there is limited knowledge on the prevalence and potential causes of IPVAW and on the potential barriers to behavioural changes.

Ultimately, the combination of the assessments made among various community members (including men, key informants and, further on, gatekeepers) will help determine the attitudes and behaviours that can be targeted by an intervention and the potential barriers to be dealt with [21,22]. The social diagnosis at hand now (i.e. that of married women) highlights widely shared opinions on which it may be essential to act to avoid a "victim-blaming" attitude in the future. This study is not a straightforward epidemiological assessment of exposure to all kinds of IPVAW. However, by asking the respondents about their own experience of non-physically moderate abusive acts like shouting and physical threats can provide some form of baseline to assess the impact of an eventual prevention program.

The study shows that, in the study area, among the women surveyed, most potential causes and triggers proposed receive considerable agreement. We interpret this as an indication that the women interviewed believe that most potential causes and triggers proposed may, at some point in a relationship, engender IPVAW and we regard this as something essential to address in the development of a prevention programme (i.e. reducing "victim blaming"). Individual-related factors are the ones that attain the highest levels of agreement. The top two potential triggers, "unpleasant verbal communication in front of others" and "unsuitable clothes in front of others" are behaviours that, in the Iranian context, are likely to put down the male partner in front of others [33]. This high consensus is very likely to be a reflection of the patriarchal – local – culture and effort will have to be put into preparing couples to address them in a non-violent manner. By contrast – and unexpectedly – despite the importance of having a male child [18,34] and of the size of the dowry in the study area, these items are among the least agreed upon as potential causes/triggers. Possible explanations for this are the fact that the women interviewed did not identify with the problem or that they did not consider it to be very prevalent in their community (in general or ethnic-specific) (more than 70% of the respondents had a male child) or social desirability bias.

As the first intervention planned for is educational, individual and relational factors will be possible to address, although in ways that are yet to be identified. Factors of that kind are among those more easily dealt with in the family. It is also known that male partners are more able to control outbursts of IPVAW than one might think [31]. For the ultimate goal of this project, life and communicational skills training could be the first step in an intervention for new couples.

These results are not easy to compare to those of other studies, as opinion surveys about potential causes of IPVAW are uncommon. One conducted in the USA reports findings consistent with the current one in that social and cultural factors were not as much mentioned as potential factors and substance abuse and men's mental problems ranked high[20]. This is echoed in the current study where, when women were asked to rank the most predisposing potential causes/triggers of husband's violence, addiction ranked first.

Furthermore, in this study, the order of agreement for potential causes, triggers or consequences of IPVAW is quite similar among the socio-demographic sub-groups and even groups of victimised women. Of the socio-demographic characteristics measured, being a housewife and being married to a low-educated husband, though not affecting the ranking of potential causes/triggers that much, does significantly push the level of agreement upwards. Even one's prior experience of moderate forms of violence has little effect on the ranking. For their part, marriage duration, number and sex of children and age have little – if any – effect on women's opinions. As suggested by others, the diversity of opinions about IPVAW can to a limited extent be accounted for by the diversity in people's background or experiences [20].

The women surveyed seem also to be very aware of the multifaceted potential consequences of IPVAW: we found very high consensus on all consequences listed, except for economic problems in the family unit.

### Strengths and limitations

The convenient sampling strategy used yielded a high response rate (94.5%), which could be attributed to the precautions taken to secure individual confidentiality [29,30] and restriction of the questions relative to victimisation. We trust that the strategy adopted also limited the sources of misclassification although victimisation may still be under-estimated [1]. The questionnaire was developed for and adapted to the study area and it was also pre-tested. The interviews were conducted on an individual basis and by three trained interviewers. During the data collection, the principal investigator had weekly meetings with the interviewers. As can be expected however, we suspect that, in spite of this, individual victimisation is under-reported [1].

As mentioned above, we interpret the high level of consensus obtained overall as an indication that the married women interviewed do consider that most potential causes and triggers of IPVAW covered may, at some point in a relationship, engender IPVAW, although not necessarily in theirs. Although confidentiality was guaranteed and the questionnaires were anonymous, a social desirability bias may have come into play. The lower acknowledgement of the absence of a male child and of the (small) size of the dowry as potentially linked to the perpetration of IPVAW might, we feel, be a case in point.

A drawback of the study is its descriptive character and the fact that the sample may not be representative of the whole target population. Let us emphasise that, for safety reasons (that primarily of the participants) [29,30], it felt inappropriate to sample and interview by household. As a consequence, by relying on health care centres, only women visiting them could be approached (80% of the target population; see methods). Though there might be an under-reporting of victimisation among the respondents, we doubt that victimisation was the one and only – perhaps even main – reason for declining participation. And, again, the non-response rate is low and there is little effect of respondents' exposure to violence on the ranking obtained as regards the potential causes and triggers of IPVAW.

Though the findings ought to be considered with caution, we believe the views reported herein may be regarded as a reasonable representation of those of married women in the target community. As there are few studies of this kind in the published literature, it remains difficult to assess how much the results are specific for those women or whether they are similar to those from other settings, in or outside the country.

## Conclusion

Our social diagnosis of women's opinions reveals that most factors proposed receive considerable agreement as potential causes and triggers of IPVAW. Although being a housewife (women's occupation) and having a relatively low-educated husband push the women's score upwards, socio-demographic characteristics and prior victimisation to non-physical violence have, in the main, little effect on the opinions of women about the causes, triggers and consequences of IPVAW. This is more so for potential causes and consequences than for potential triggers. Yet, the highest consensus is attained on potential triggers that tend to have a humiliating effect on a husband in front of other people. These results reveal attitudes and behaviours that need thorough consideration in the development of an educational programme where both acceptance of the victim responsibility and perpetration by the partner of violence acts will need to be dealt with.

## Competing interests

The authors declare that they have no competing interests.

## Authors' contributions

All three authors contributed to the conception of the study design and data collection instrument. BH supervised and coordinated the data collection, entered and analysed the data, and co-authored the manuscript. MGF participated in the finalisation of the manuscript. LL took part in the data analysis and the writing-up of the manuscript. All authors read and approved the final manuscript.

## Pre-publication history

The pre-publication history for this paper can be accessed here:


